# Mapping Spatial Variability of Soil Salinity in a Coastal Paddy Field Based on Electromagnetic Sensors

**DOI:** 10.1371/journal.pone.0127996

**Published:** 2015-05-28

**Authors:** Yan Guo, Jingyi Huang, Zhou Shi, Hongyi Li

**Affiliations:** 1 Institute of Agricultural Remote Sensing and Information Technology Application, College of Environmental and Resource Sciences, Zhejiang University, Hangzhou, China; 2 Institute of Agricultural Economics and Information, Henan Academy of Agricultural Sciences, Zhengzhou, China; 3 School of Biological, Earth and Environmental Science, The University of New South Wales, Kensington, NSW 2052, Australia; 4 School of Tourism and Urban Management, Jiangxi University of Finance and Economics, Nanchang, China; University of Vigo, SPAIN

## Abstract

In coastal China, there is an urgent need to increase land area for agricultural production and urban development, where there is a rapid growing population. One solution is land reclamation from coastal tidelands, but soil salinization is problematic. As such, it is very important to characterize and map the within-field variability of soil salinity in space and time. Conventional methods are often time-consuming, expensive, labor-intensive, and unpractical. Fortunately, proximal sensing has become an important technology in characterizing within-field spatial variability. In this study, we employed the EM38 to study spatial variability of soil salinity in a coastal paddy field. Significant correlation relationship between EC_a_ and EC_1:5_ (i.e. r >0.9) allowed us to use EM38 data to characterize the spatial variability of soil salinity. Geostatistical methods were used to determine the horizontal spatio-temporal variability of soil salinity over three consecutive years. The study found that the distribution of salinity was heterogeneous and the leaching of salts was more significant in the edges of the study field. By inverting the EM38 data using a Quasi-3D inversion algorithm, the vertical spatio-temporal variability of soil salinity was determined and the leaching of salts over time was easily identified. The methodology of this study can be used as guidance for researchers interested in understanding soil salinity development as well as land managers aiming for effective soil salinity monitoring and management practices. In order to better characterize the variations in soil salinity to a deeper soil profile, the deeper mode of EM38 (i.e., EM38v) as well as other EMI instruments (e.g. DUALEM-421) can be incorporated to conduct Quasi-3D inversions for deeper soil profiles.

## Introduction

Over the past decades, much of the tidelands in China have been reclaimed for agriculture and urban buffer zone [1]. However, the highly saline coastal soil often causes adverse effects on agricultural productivity, particularly in the first 20 years of agricultural production. After nearly 20 years of farming, the soil salinity has changed dramatically and the salts have leached into deeper soil profiles due to the irrigation farming and high level of precipitation across these coastal areas. In order to make profits from the reclaimed soils, farmers start to plant more profitable crops (e.g. rice) on the reclaimed lands. However, loss of yield often occurs because of the subjective diagnose of the salinity level of the reclaimed soils using farmers’ experiences. In order to better management the reclaimed soils (especially salinity) within these coastal areas, it is important to characterize the spatial variations of soil salinity, especially within the root zone [2], in an accurate and efficient way.

Conventional soil salinity mapping has been done by visual observations with limited laboratory measurements (United States Salinity Laboratory Staff, 1954; Soil Survey Division Staff, 1993). However, visual observations provide only qualitative information [3] and laboratory methods are often time-consuming, expensive, and labor-intensive [4]. In order to quantify the soil variability using geostatistical methods, approximately 100 sample points are required to estimate a spatial statistical model [5]. For example, in an attempt to map soil salinity in a field in Southern Alberta Gallichand et al. [6] collected 80 soil samples at two different depths on a regular grid and used 2D- and 3D-kriging to interpolate the conductivity of the saturated paste extract (i.e., EC_e_) of the study area.

The need for rapid, reliable, and easy-to-take measurements of soil salinity at field and landscape scales gave birth to the proximal sensing electromagnetic induction (EMI) [4]. EMI can produce a large number of georeferenced and quantitative measurements that can be easily correlated with the spatial variability of salinity [3]. The most commonly used conductivity meter (EM38, Geonics Ltd., Ont, Canada) measures soil apparent electrical conductivity (EC_a_). The EM38 also has been used to map soil properties (e.g., EC_e_ and soil moisture) using various calibration models [7–12] at field [10–11], region [13], and catchment [14] scales.

In addition to mapping the horizontal variability of soil salinity, a number of researchers have attempted to measure EC_a_ at different depths with an inversion algorithm. A pioneering work in this field was undertaken by Hendrickx et al. [15]. They used Tikhonov regularization to invert the EM38 data using measurements collected at different heights above the ground and in different directions. Though successful, the inversion was essentially a 1-Dimension inversion and could not reflect the lateral variation of soil salinity. Several years later, researchers developed 2-Dimension inversion algorithms to invert the EC_a_ data onto 2-D vertical slices and 2-D horizontal slices [16–18]. Most recently, a combination of vertical slices and horizontal slices was utilized to determine the 3-D variability of soil conductivity [19–21]. With these inversion approaches, spatial variability of soil electrical conductivity and the correlated soil properties (e.g. salinity) can be presented in a 2-D or 3-D view.

Despite the successful application of EMI in soil salinity mapping [19, 22–24], few studies have reported the use of EMI to determine temporal variability of soil salinity from a multi-dimensional view. The objective of this study is to map the spatio-temporal variability of soil salinity in a reclaimed costal paddy field using three years of EM38 data. Geostatistical analysis and a Quasi-3D inversion algorithm were combined to map the horizontal and vertical spatio-temporal variability of soil salinity in the study field.

## Materials and Methods

### Ethics Statement

We randomly chose 3 coastal paddy fields in the northern region of Shangyu City, Zhejiang Province, southeast of Hangzhou Bay, China and got permission from Agricultural Bureaus of Shangyu. One field (4.25 ha) was used to collect EM data in three consecutive years, and the others were employed to collect validation data. These fields were the experimental fields in Zhejiang University. No endangered or protected species were involved in the study.

### Study Area

**Fig 1:**
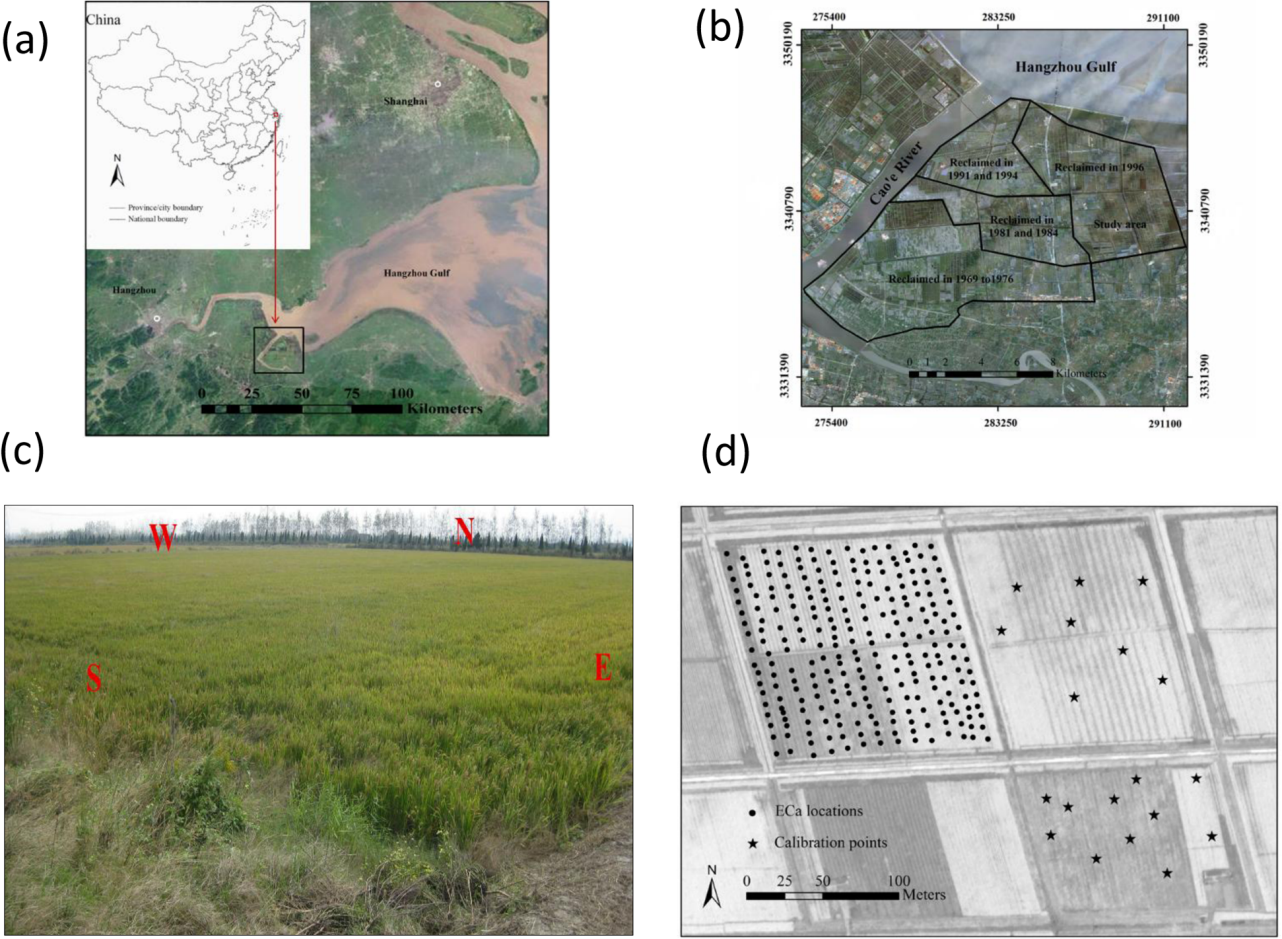
Locations of the study field and EC_a_ measurements. (a) Location of the study field with reference to the Hangzhou Gulf, (b) reclaimed lands over the past 40 years; (c) A photo of the study field (Taken on October 2010) and (d) Location of EC_**a**_ measurements in 2009 across the study area.

The study was conducted in a coastal saline area located in the northern region of Shangyu City, Zhejiang Province, southeast of Hangzhou bay, China. The climate is subtropical with an average annual temperature of 16.5°C. The average daily maximum and minimum monthly temperatures are 4°C (January) and 28°C (July), respectively. Average annual precipitation is 1300 mm, with the heaviest rainfall occurring during two rainy seasons between March and June and also during September. Over the past 40 years, approximately 17 000 ha of coastal land has been reclaimed around Shangyu City in successive programs (Fig 1A and 1B). The investigated fields were reclaimed in 1996. They were separated by small embankments (bunds) which ensured flooded conditions within each of the study fields. A photo of the study field is shown in Fig 1C.

### Data Collection and Processing

In the present study, an EM38proto (Geonics, Ltd., Ont., Canada) was used to record EC_a_ data with a Geonics EM38 Data Logging System (DAS70-CX) and a field computer (Allegro CX). A separate Global Positioning System (GPS) with differential correction within 2 m was used for geo-reference.

In order to understand the correlation between soil salinity and EC_a_ data, a preliminary EM38 survey was conducted in the adjacent fields to the east and southeast of the study field (Fig 1D). Afterwards, 19 soil cores were collected in these fields (Fig 1D) to a depth of 80 cm with a 20-cm interval (i.e. 0–20, 20–40, 40–60, 60–80 cm). The soils cores were selected to cover the different values of EC_a_ measurements, including high (i.e. EC_a_≥300 mS/m), medium (i.e. 200 mS/m<EC_a_<300 mS/m) and low (i.e. EC_a_≤200 mS/m) values. EC_a_ measurements were recorded in the vertical (EM38v) and horizontal (EM38h) modes, respectively. Soil samples at various soil depths (i.e. 0–20 cm, 20–40 cm, 40–60 cm, 60–80 cm) were collected for lab analysis of soil salinity by a conventional conductivity meter using a 1:5 soil: water suspension (EC_1:5_). Detailed information can be found in S1 File.

Subsequently, EC_a_ measurements were taken along an approximate 20 m grid along the furrows in the study field (northwest of Fig 1D) in three consecutive years. There were 251, 256, and 339 EC_a_ measurements collected in October 2009, November 2010, and November 2011, respectively. In order to calculate the coefficient of variation over time, EM38 measurements in 2010 and 2011 were harmonized onto a common grid consisting of the 251 EC_a_ measurement sites in 2009 (See Fig 1D) using the nearest neighbor algorithm available in ArcGIS 9.3 (ESRI, 2013).

The EC_a_ measurements were taken after the rice was harvested and the field was drained. According to Robinson et al. [25], EM38 measurements drift significantly when temperatures are over 40°C and the drift is more obvious for small EC_a_ readings (i.e., less than 100 mS/m). Because the temperature conditions were similar when the three surveys were taken (approximately 25°C) and the study area was highly conductive, we did not calibrate the EC_a_ measurements to a standard temperature of 25°C as suggested by Sheets and Hendrickx [26]. However, EM38 was calibrated according to the manual before the surveys to reduce the error [27–28].

### Characterizing Horizontal Spatio-Temporal Variability of Soil Salinity using Geostatistical Approaches

Geostatistical approaches are often used to define the variance structure, spatial distribution, and trend changes of soil properties. Ordinary kriging (OK) is one of the most popular interpolation methods [29]. The OK method uses a semivariogram to quantify the spatial variation of a regionalized variable [30]:

$$\gamma \left(h\right)=\frac{1}{2N\left(h\right)}\overset{N(h)}{\underset{i=1}{\sum }}[Z\left(x_{i}\right)\text{-}Z{\left(x_{i}+h\right]}^{2})$$ (1)

where *γ*(*h*) is a semivariogram that measures the mean variability between two points *x* and *x* + *h* as a function of their distance *h*; *Z*(*x*_*i*_) and *Z*(*x*_*i+h*_) are the values of the variable *Z* at location *x*_*i*_ and *x*_*i*_+*h*; *N*(*h*) is the number of pairs of sample points separated by the lag distance *h*.

EC_a_ measurements were interpolated by OK using Eq (2) [30] with ArcGIS 9.3 (ESRI, 2013) to calculate the horizontal spatial variability of soil salinity:

$$Z^{*}\;\left(x_{0}\right)=\overset{n}{\underset{i=1}{\sum }}\lambda_{i}\;Z(x_{i})$$ (2)

where *Z* ^*^(*x*_*0*_) is the predicted EC_a_ at location *x*_0_; *Z*(*x*_*i*_) is the measured EC_a_ at location *x*_*i*_; *λ*_*i*_ is the weight assigned to the observation Z(*x*_*i*_); and *n* is the number of measurements.

With regard to the horizontal temporal variability, we calculated the coefficient of variation (*CVt*_*i*_) over time at each measurement site to assess the stability of soil salinity (Eq 3). The technique has been used by Blackmore [31] to characterize the temporal stability of crop yields and by Shi et al. [32] to assess the stability of soil properties in grasslands.

$$CVt_{i}=\frac{\sqrt{\left(n\times \sum_{t=1}^{n}{\text{ECa}}_{it}^{2}\text{-}(\sum_{t=1}^{n}{\text{ECa}}_{it}^{2}))/n\times n(n\text{-}1\right)}}{(\sum_{t=1}^{n}{\text{ECa}}_{it}^{2})/n}$$ (3)

Where *CVt*_*i*_ is the coefficient of variation over three years at the *i*th ECa measurement site in the *t*th year and *n* is the number of EC_a_ measurements.

### Mapping Vertical Spatio-Temporal Variation in Soil Salinity Using Quasi-3D Inversion

In order to determine the distribution of true electrical conductivity (σ—mS/m) at different depths from the EC_a_ measurements, an inversion software (EM4Soil) was used to convert EC_a_ to σ. We employed the Quasi-3D module (Q3Dm) of the software following the procedure of Monteiro Santos et al. [33] to invert the EC_a_ data of the three consecutive years. Q3Dm is a 1-Dimensional Spatial Constrained technique (1-D SCI) and a forward modeling approach. It assumes that below each measured site the 1-Dimension variation of the soil conductivity is constrained by the variation under neighboring sites. The modeling process is based upon the cumulative function [27]. The inversion algorithm is based on the Occam regularization method [34–35].

As the software requires standard grid files for inversion, gridding was applied onto the raw data set using the Gridding Tool of the Q3Dm package. The gridding was based on the inverse distance weighted method (EM4SOIL Manual, 2011). In this study, a weight value of 2.0 was selected and the grid consisted of 10 x-lines (west-east) and 8 y-lines (south-north) with grid spacing of 18 m.

After gridding, inversion of EC_a_ data was performed using Algorithm 3 (designed for inversion of electromagnetic induction signal from single sensor) with a damping factor of 0.3, 10 iterations, a data error of 1.00, and a misfit target of 0.20. An initial 2-layer laterally homogeneous model was predefined with initial electrical conductivity of 10 mS/m for both layers, a depth of 0.6 m for first the upper layer, and a depth of 1.2 m for the bottom layer. EC_a_ data of the three consecutive years were inverted separately.

## Results and Discussion

### Calibration of EM38 Data using Soil EC_1:5_

**Fig 2:**
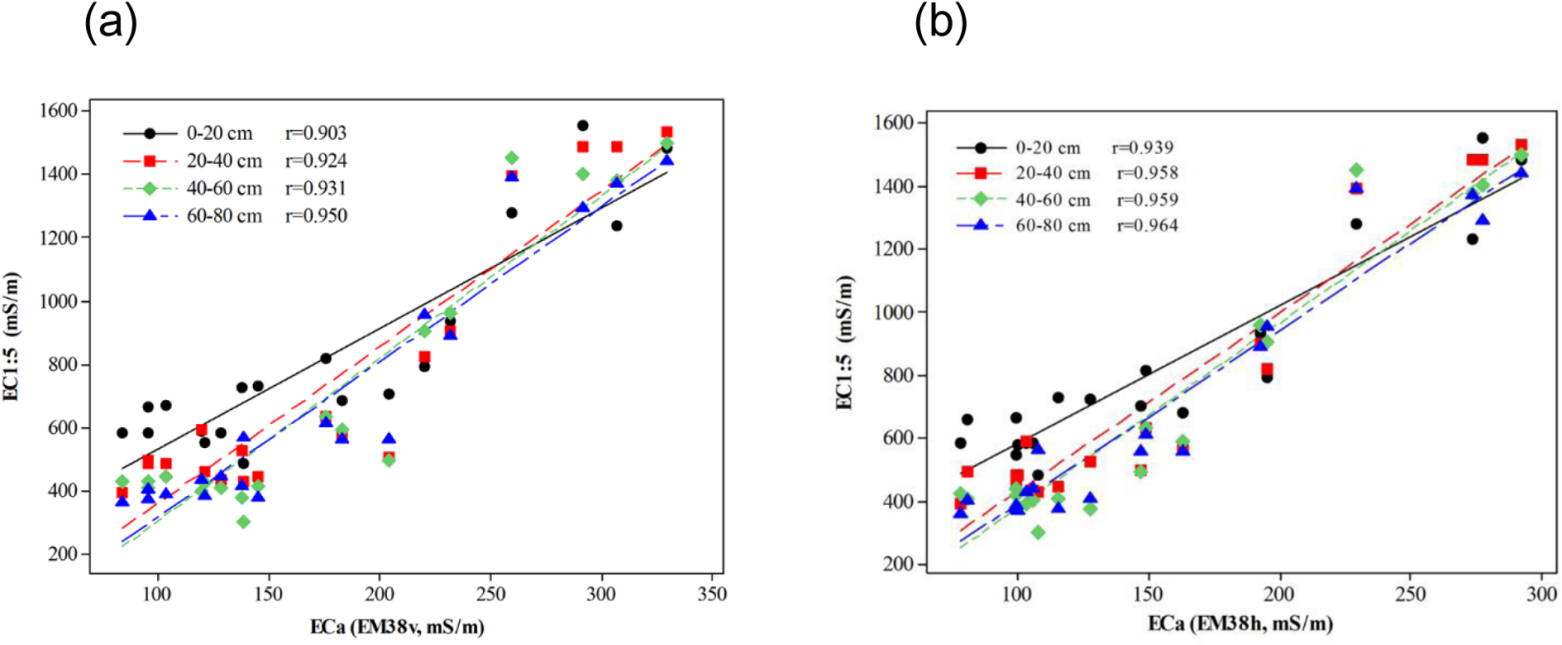
Pearson correlation coefficients between soil EC_1:5_ (mS/m) of the calibration points at various depths and EC_a_ (mS/m) measurements with regard to EM38v (a) and EM38h (b).

In this study, soil EC_1:5_ was used as an indicator of soil salinity. Fig 2 shows the Pearson correlation coefficients between measured EC_1:5_ and EC_a_ from both EM38v and EM38h. Significant correlations are found between EC_1:5_ and EC_a_ for both EM38v and EM38h at different depths (r >0.90, P < 0.001). According to Li et al. [19], the variation of soil salinity in the adjacent field of our study area was successfully characterized with EC_a_ measurements from EM38h. Therefore, it was assumed that the variations of soil salinity (i.e. EC_1:5_) in our study area can be solely explained by the variations of EC_a_ values. Additionally, it is worth noting that the correlations between EC_1:5_ and EC_a_ from EM38h are higher than those between EC_1:5_ and EC_a_ from EM38v. Because the effective measuring depth of EM38h is approximately the rootzone, which is of great importance for studying the leaching process of salt, EC_a_ measurements from EM38h were collected in the following years (i.e. 2009, 2010 and 2011) and used for characterization of spatio-temporal variations of soil salinity in the study area.

### Statistical Analysis of Multi-Temporal EM38 Data

Table 1 shows basic summary statistics of measured EC_a_ in 2009, 2010, and 2011. The average values decrease substantially from 2009 (166.19 mS/m) to 2010 (134.02 mS/m) and 2011 (113.29 mS/m). Similarly, the quartile estimates of EC_a_ show a decreasing trend from 2009 to 2011. The Shapiro-Wilk statistics are 0.925, 0.930 and 0.925 with P-values less than 0.01, which indicate significant deviation from normality. In such cases, Box-Cox transformation method was used to transform the data by a monotonically increasing (or decreasing). In the next section, the datasets were normalized by this method.

**Table 1:** Descriptive statistics of EC_a_ (mS/m) in 2009, 2010 and 2011.

Year	n	Mean	Stde	Min	25%	Median	75%	Max	Shapiro-Wilk Test
2009	251	166.19	3.50	51.3	145.4	179.3	195.6	226.7	0.925
2010	256	134.02	2.00	20.1	96.15	151.85	182.9	217.7	0.930
2011	339	113.29	3.01	10.5	73.2	140.2	157.9	181.8	0.925

**Fig 3:**
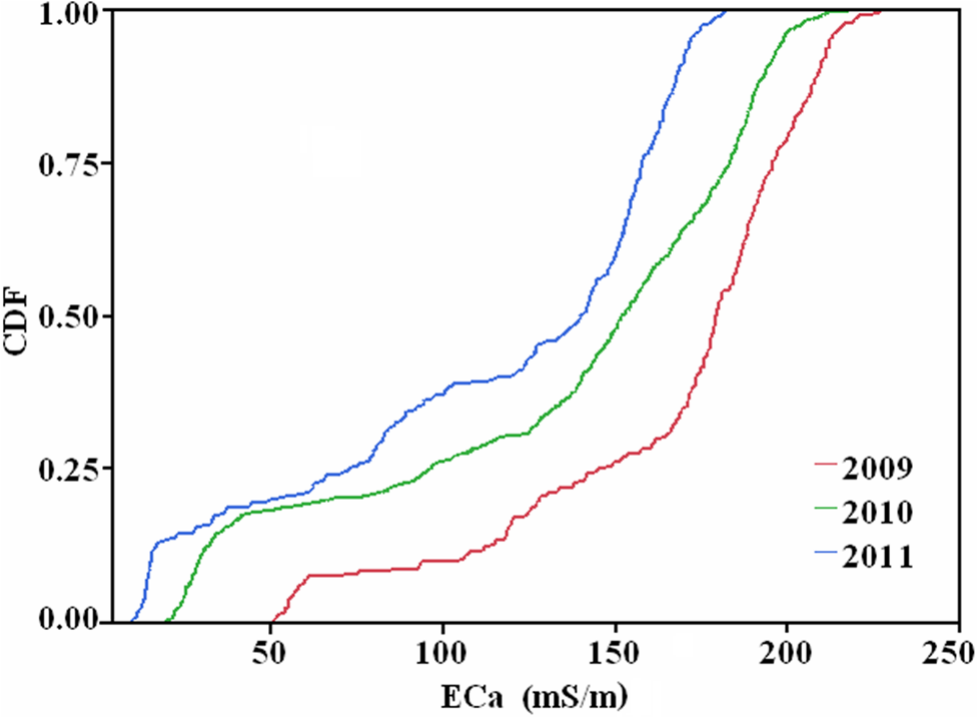
Plot of cumulative distribution function (CDF) of EC_a_ (mS/m) in 2009, 2010 and 2011.

Fig 3 shows the curves of the cumulative distribution function (CDF) for the study area which illustrates visible temporal variations of soil salinity among the three years. For a given EC_a_ value, CDF is largest in 2011 and smallest in 2009. In order to quantify the difference, we used the Tukey-Kramer multiple comparison procedure. The values listed in Table 2 are the actual absolute differences in the means minus the least significant difference (i.e., abs-LSD). Positive abs-LSD values in Table 2 indicate significant difference (P < 0.01). It was found that the most significant change of EC_a_ occurred between 2009 and 2011, followed by the period from 2009 to 2010, and then between 2010 and 2011.

**Table 2:** Comparison ofmeans of EC_a_ (mS/m) for 2009, 2010, and 2011 using the Tukey-Kramer test.

Year	Mean	2009	2010	2011
2009	166.19	-11.61	22.71	42.06
2010	134.02		-6.64	12.24
2011	113.29			-9.99

### Characterizing Horizontal Spatio-Temporal Variability of Soil Salinity Using Geostatistical Approaches

**Fig 4:**
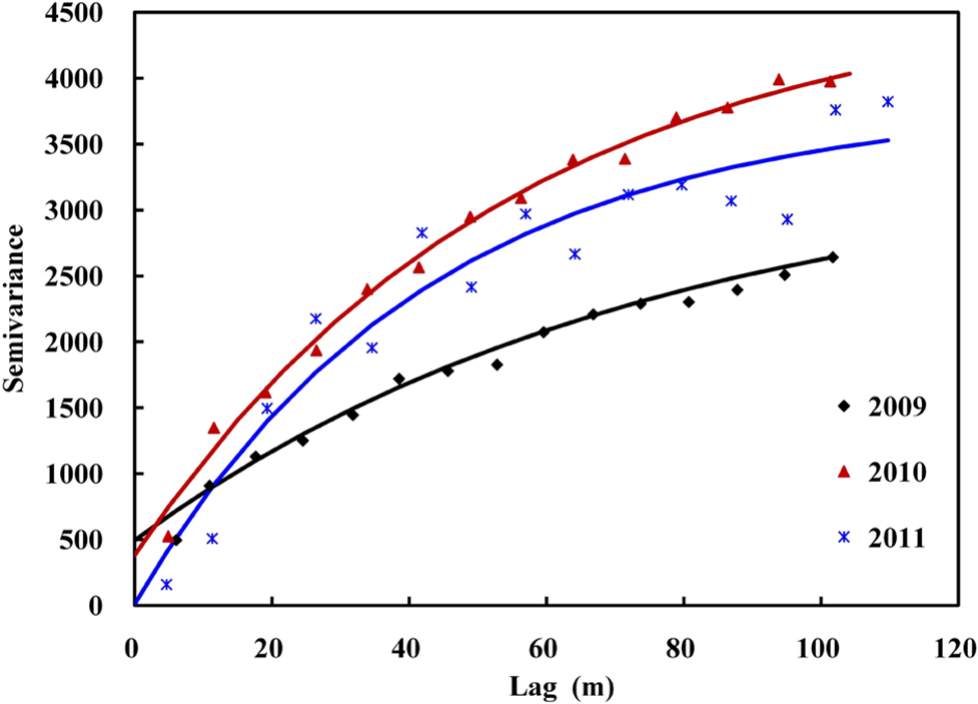
Semivariance and fitted models (solid lines) for soil EC_a_ (mS/m) from 2009 to 2011.

Fig 4 shows the plot of experimental semivariances and the fitted semivariogram models for the EC_a_ from 2009 to 2011. The parameters of these models are shown in Table 3. The semivariances of the models indicate that the spatial behavior has good continuity in space and can be modeled quite well with exponential models. However, different tendencies were found for models of the three years. The nugget value (C_0_) decreases from 2009 to 2011, indicating that the variations of soil salinity over a short distance have become smaller and smaller. The ratios of C_0_ to sill (C+C_0_) decline sharply from 17.07% (2009) to 0.26% (2011). According to Shi et al. [36], a ratio less than 0.25 indicates strong spatial dependence; a value between 0.25 and 0.75 denotes moderate spatial dependence; and a value greater than 0.75 indicates weak spatial dependence. In this regard, we can conclude that the spatial autocorrelation of EC_a_ was becoming stronger during the study period. This increase may be caused by the alternating irrigation and drainage practices necessary for rice cultivation. In addition, the relatively large nugget effect in the EC_a_ data is most probably the consequence of an uneven distribution of soil salinity between ridge and furrow irrigation, perhaps associated with a small georeferencing error; Also the abrupt transitions in soil salinity, i.e. a short distance variability not to taken into account by the density of the sampling, and in this case, the nugget effect decreases, it can be assumed that the transitions, initially steep, soften between 2009 and 2011.

**Table 3:** Models and parameters of semivariogram for ordinary kriging of soil EC_a_ (mS/m) in 2009, 2010 and 2011.

Year	Semivariogram model	Nugget (C_0_)	Sill (C_0_+C)	C_0_/(C_0_+C)	Range (A)	r^2^
2009	Exponential model	495	2899	17.07	225.90	0.964
2010	Exponential model	380	4302	8.83	165.00	0.912
2011	Exponential model	10	3807	0.26	127.50	0.928

**Fig 5:**
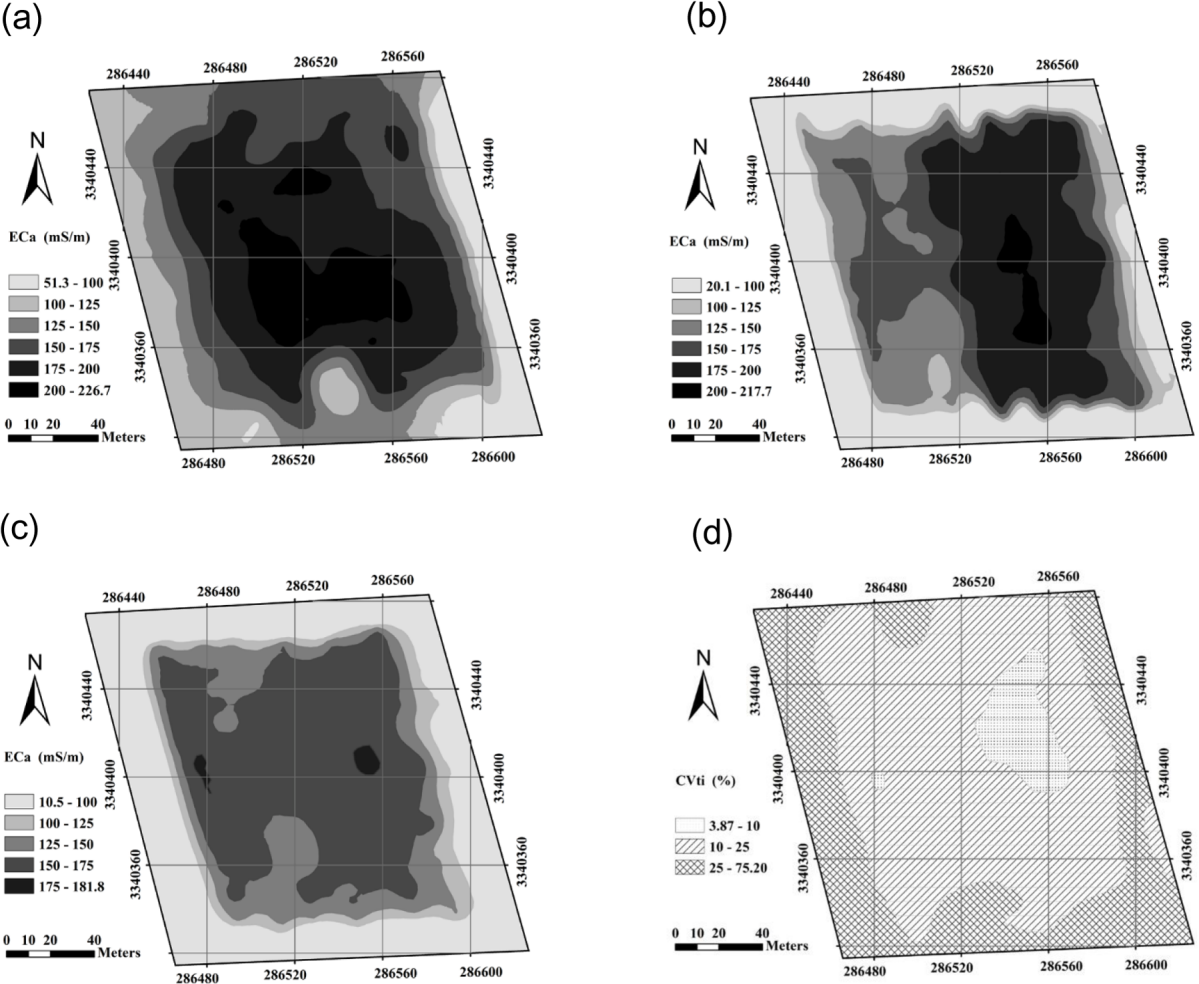
Spatial distribution of soil EC_a_ (mS/m) in (a) 2009, (b) 2010, and (c) 2011. The plot of (d) coefficient of variation (*CVti*) over three years.

Maps of EC_a_ in 2009, 2010, and 2011 generated by ordinary kriging are shown in Fig 5A, 5B and 5C, respectively. These maps show that EC_a_ has decreased over the three years. For example, in a central block of the field (Easting: 286,520 m—286,560 m; Northing: 3,340,360 m—3,340,400 m) EC_a_ was mostly larger than 200 mS/m in 2009, but the values decreased to175–200 mS/m in 2010 and then dropped to 125–150 mS/m in 2011.The decreasing ECa was most likely due to the irrigation and drainage practices for rice cultivation which leached the salts into a deep soil profile or the ground water.

The spatial distribution of soil salinity also changed. In 2009, the largest EC_a_ values (> 200 mS/m) were found in the center of the field and values decreased with distance from the center. However, in 2010 the largest EC_a_ values (> 200 mS/m) were found in the right half of the field and there was a distinctive difference in EC_a_ between the left and right halves of the field. With regard to year 2011, any differences in EC_a_ between the left and right field halves were not obvious and the field was mostly dominated by EC_a_ values of 125–150 mS/m. The heterogeneous and changing salinity distribution of the study area may be caused by the presence of ditches in the study area. Because the study area is a paddy field and surrounded by ditches (Fig 1C and 1D), it will be fully saturated with water during the crop cultivation, especially continuously irrigation and drainage made the salt wash away along with water in rice growth. Therefore, the rate of leaching of salts will be a function of the distance to the ditches. This is consistent with the large coefficient of variation (*CVt*_*i*_) values in the margins of the study area shown in Fig 5D.

In order to quantify the temporal stability of salinity, *CVt*_*i*_ values of each EC_a_ measurement over three years are shown in Fig 5D. According to Shi et al. [36], the variation should be considered stable when *CVt*_*i*_ is less than 10%, moderately stable when *CVt*_*i*_ is between 10% and 25%, and unstable when *CVt*_*i*_ is larger than 25%. Interestingly, the area with a high salinity content (Easting: 286,520 m—286,560 m; Northing: 3,340,400 m—3,340,440 m) displays temporal stability, while the surrounding area shows temporal instability, especially the edges of the field with a lower salinity level. This is consistent with the reports by Shi et al. [36]. The sharp change of salinity within the field edges may be due to the presence of irrigation ditches around the field where large amounts of irrigation water allow salts to leach into deeper soils.

### Mapping Vertical Spatio-Temporal Variation in Soil Salinity Using Quasi-3D Inversion

**Fig 6:**
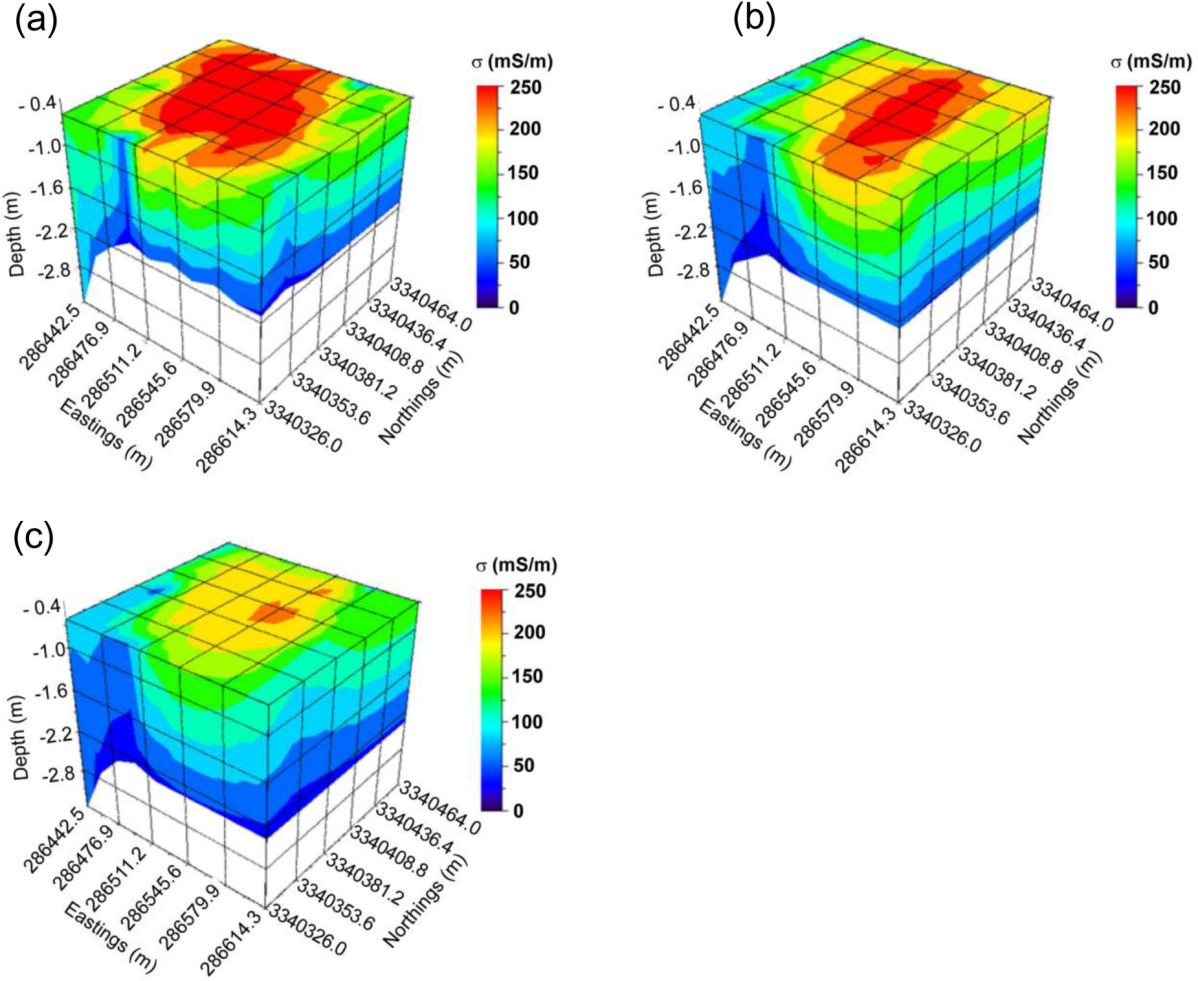
Quasi-3D models of soil electrical conductivity (mS/m) in (a) 2009, (b) 2010 and (c) 2011 across the study area.

The Quasi-3D inversion results are shown in Fig 6. The vertical spatio-temporal variation of the soil salinity can be explained by the distribution of modeled σ. The soils in the study area were predicted to be inverted salinity profiles for all the three years. It was consistent with the calibration results shown in Fig 2. The widely distributed inverted salinity profiles were mostly likely caused by irrigation farming and high level of precipitation. Viewing from the 2-D cross-section oriented west-east, the salts of the soil migrate downwards over the three years. For example, the area with Eastings from 286,511.2 m to 286,545.6 m and depths from 0.5 m to 1.0 m was primarily dominated by σvalues between 150–200 mS/m in 2009. However, the conductivity of the area decreased to 100–175 mS/m in 2010. Furthermore, in the year of 2011 this conductivity was mostly between 100–125 mS/m. The decreasing distribution of soil salinity is also evident in 2-D cross-sections of the Quasi-3D models oriented south-north. The phenomenon is consistent with the vertical distribution of the soil salinity of paddy fields, whereby the salts will be washed out from the root zone over years of cultivation [19, 33]. The vertical distribution of salinity is also consistent with the annual precipitation of the study area (i.e., 1,300 mm). Additionally, the horizontal 2-D cross-sections at the top of the models for the three years are consistent with the kriging maps shown in Fig 5. This implies that the two approaches for determining spatio-temporal variation of salinity are reliable and consistent with each other.

## Conclusions

Repeated electromagnetic induction (EMI) surveys were carried out across a reclaimed paddy field in coastal regions of China over a three-year period. Significant correlation between apparent electrical conductivity (EC_a_) and soil EC_1:5_ (r > 0.9, P < 0.001) allowed for rapid characterization of the spatio-temporal variation in soil salinity using EC_a_ data. Ordinary kriging of EC_a_ data showed the horizontal distribution of soil salinity was heterogeneous and the decrease of salinity may be a function of the distance to the irrigation ditches. Using a quasi-3D inversion approach, soils in the study areas were predicted to be inverted salinity profiles. The vertical leaching of salts with time was also successfully mapped, which was consistent with the location of irrigation ditches and high precipitation.

It is concluded that spatio-temporal variability of soil salinity in paddy fields can be characterized by the cost-effective and efficient EMI surveys. The methodology of this study can be used as guidance for researchers interested in understanding soil salinity development as well as land managers aiming for effective soil salinity monitoring and management practices. In order to better characterize the variations in soil salinity to a deeper soil profile, the deeper mode of EM38 (i.e., EM38v) as well as other EMI instruments (e.g. DUALEM-421) can be incorporated to conduct Quasi-3D inversions for deeper soil profiles [21, 37].

## Supporting Information

S1 File EC_a_ measurements were harmonized onto a common grid consisting of the 251 EC_a_ measurement sites in 2009 using the nearest neighbor algorithm available in ArcGIS 9.3 (ESRI, 2013). 19 soil calibration points were sampled with recorded EC_a_ measurements for the vertical (EM38v) and horizontal (EM38h) modes, respectively.
(XLSX)
